# Pharmacokinetics of Cannabidiol, Cannabidiolic Acid, Δ9-Tetrahydrocannabinol, Tetrahydrocannabinolic Acid and Related Metabolites in Canine Serum After Dosing With Three Oral Forms of Hemp Extract

**DOI:** 10.3389/fvets.2020.00505

**Published:** 2020-09-04

**Authors:** Joseph J. Wakshlag, Wayne S. Schwark, Kelly A. Deabold, Bryce N. Talsma, Stephen Cital, Alex Lyubimov, Asif Iqbal, Alexander Zakharov

**Affiliations:** ^1^Department of Clinical Sciences, Cornell University College of Veterinary Medicine, Ithaca, NY, United States; ^2^Department of Molecular Medicine, Cornell University College of Veterinary Medicine, Ithaca, NY, United States; ^3^University of Florida Comparative Diagnostic and Population Medicine, Gainesville, FL, United States; ^4^Ellevet Sciences, Product Development and Scientific Communications, Portland, ME, United States; ^5^Toxicology Research Laboratory, Department of Pharmacology, College of Medicine, University of Illinois at Chicago, Chicago, IL, United States

**Keywords:** dog, hemp, cannabidiol, cannabidiolic acid, tetrahydrocannabinol, tetrahydrocannabinolic acid, pharmacokinetics

## Abstract

Cannabidiol (CBD)-rich hemp extract use is increasing in veterinary medicine with little examination of serum cannabinoids. Many products contain small amounts of Δ9-tetrahydrocannabinol (THC), and precursor carboxylic acid forms of CBD and THC known as cannabidiolic acid (CBDA) and tetrahydrocannabinolic acid (THCA). Examination of the pharmacokinetics of CBD, CBDA, THC, and THCA on three oral forms of CBD-rich hemp extract that contained near equal amounts of CBD and CBDA, and minor amounts (<0.3% by weight) of THC and THCA in dogs was performed. In addition, we assess the metabolized psychoactive component of THC, 11-hydroxy-Δ9-tetrahydrocannabinol (11-OH-THC) and CBD metabolites 7-hydroxycannabidiol (7-OH-CBD) and 7-nor-7-carboxycannabidiol (7-COOH-CBD) to better understand the pharmacokinetic differences between three formulations regarding THC and CBD, and their metabolism. Six purpose-bred female beagles were utilized for study purposes, each having an initial 7-point, 24-h pharmacokinetic study performed using a dose of 2 mg/kg body weight of CBD/CBDA (~1 mg/kg CBD and ~1 mg/kg CBDA). Dogs were then dosed every 12 h for 2 weeks and had further serum analyses at weeks 1 and 2, 6 h after the morning dose to assess serum cannabinoids. Serum was analyzed for each cannabinoid or cannabinoid metabolite using liquid chromatography and tandem mass spectroscopy (LC-MS/MS). Regardless of the form provided (1, 2, or 3) the 24-h pharmacokinetics for CBD, CBDA, and THCA were similar, with only Form 2 generating enough data above the lower limit of quantitation to assess pharmacokinetics of THC. CBDA and THCA concentrations were 2- to 3-fold higher than CBD and THC concentrations, respectively. The 1- and 2-week steady-state concentrations were not significantly different between the two oils or the soft chew forms. CBDA concentrations were statistically higher with Form 2 than the other forms, showing superior absorption/retention of CBDA. Furthermore, Form 1 showed less THCA retention than either the soft chew Form 3 or Form 2 at weeks 1 and 2. THC was below the quantitation limit of the assay for nearly all samples. Overall, these findings suggest CBDA and THCA are absorbed or eliminated differently than CBD or THC, respectively, and that a partial lecithin base provides superior absorption and/or retention of CBDA and THCA.

## Introduction

The use of cannabidiol (CBD)-rich hemp-extract supplements is increasing in companion animal medicine with very few publications on the efficacy of these products in clinical conditions. To date, there are only three clinical publications with positive outcomes in canine osteoarthritis and epilepsy (1–3). Two publications have also examined the pharmacokinetics of similar dosing at 2 mg/kg of cannabinoids from hemp products (1, 4). Both the osteoarthritis study BID dosing of 2 and 2.5 mg/kg in the seizure study were used, while the third study was a non-placebo controlled study showing efficacy in a dose escalation once per day of cannabinoids between 1.5 and 2.5 mg/kg, with CBD representing the primary cannabinoid in this hemp extract study (1–3). Further work on absorption at the 2 mg/kg dose using a CBD and cannabidiolic acid (CBDA) mixture at ~50% of each in a soft chew form [maximum serum concentration (Cmax) ~300 ng/mL CBD] showed superior absorption when compared to prior data using an oil base (Cmax ~100 ng/mL CBD), providing some evidence along with others that dosing with food may be advantageous (4, 5).

With the measurement of serum CBD now published in canines conjures a presumption that CBDA may undergo biotransformation to CBD based on a single human study (6). *In-vitro* systems of gastric absorption suggest that CBDA might become CBD in the gastrointestinal tract and that CBD has the potential to become Δ9-tetrahydrocannabinol (THC) through either gastric or hepatic conversion (7, 8). Currently, there is little evidence of this occurrence *in vivo*, but in general it is thought that CBD does not become THC *in vivo* (9). Conversely, literature has shown that providing oral CBDA rather than CBD, actually results in a 3-fold higher Cmax of CBD in the bloodstream of people (6). This has led to the idea that CBDA may be a more bioavailable cannabinoid that becomes CBD *in vivo* or that it might help with the absorption of CBD, allowing for higher serum concentrations with lower dosing (6). To date, there has been little examination of serum CBDA in any species to prove this postulation.

In conjunction with using CBDA to improve absorption or retention of CBD, current literature is suggesting that CBD absorption is enhanced by 3- to 5-fold when providing it in conjunction with a fatty meal (5, 10–14). The current recommendations for Epidiolex, the FDA-approved human CBD product, is that it be fed with a meal to enhance absorption (12). Our prior publication in dogs showed that providing CBD in a soft chew food matrix improved absorption with higher CBD maximal concentrations providing further evidence that a food matrix might improve absorption but may lower retention times and half-life times in dogs (4).

Nearly all of the hemp-related products being utilized in the supplement market, including some labeled as CBD isolates, have varying levels of THC and tetrahydrocannabinolic acid (THCA), and although the THC and THCA levels are often below 0.3% by weight, there may be absorption of these cannabinoids, which are psychotropic and neuroprotective, respectively (15–17). The idea that THC may become 11-hydroxy-Δ9-tetrahydrocannabinol (11-OH-THC), the primary intoxicating form, in significant amounts enabling it to induce clinical signs in dogs is concerning. Only preliminary research has been done evaluating THC and 11-OH-THC concentrations in dogs being given oral hemp extracts, with none evaluating THCA forms (17). This concern, in line with the known enhanced uptake of THC with food in general, creates a real conundrum for veterinarians concerning the safety of providing small amounts of THC and its derivatives when using complete spectrum hemp plant extracts (12, 18). In addition, prior clinical studies have identified rises in hepatic enzymes, more specifically ALP, during oral hemp extract administration; and in normal healthy dogs being provided 10 mg/kg and above of CBD or CBD-rich hemp extracts (1, 2, 19). Further clarity is necessary regarding hepatic effects during typical dosing regimens currently being used.

The aim of our study was 4-fold: (1) provide pharmacokinetic information on a hemp extract with known CBD and CBDA concentrations and to examine whether low THC and THCA concentrations could be detected when fed in three oral food-based forms using 24-h pharmacokinetic profiling as well as 1- and 2-week average serum concentrations; (2) elucidate the gastrointestinal absorption kinetics of CBDA and potentially THCA; (3) determine whether we could detect 11-OH-THC, the major intoxicating metabolite of THC, and 7-OH-CBD and 7-COOH-CBD as primary metabolites of CBD in human literature; and (4) examine the serum biochemical indicators of hepatic function before and after 2 weeks of twice-daily dosing to ensure that the liver is not affected by these particular forms at the 2 mg/kg dosing regimen.

## Materials and Methods

### Dogs

Six purpose-bred intact female beagles between the ages of 14 and 18 months were acquired and housed under an Institutional Animal Care and Use Committee approved protocol at the University of Florida (2019-0024). The dogs were acclimated to the environment for 2 weeks and had initial bloodwork and physical examinations done by the Animal Care Service Veterinarians before beginning the study. All of the dogs were between 8.4 and 9.7 kg for the duration of the study. The dogs were weighed weekly to assess relative dosing with the three different forms of an oral cannabinoid-rich hemp product. After each 2-week phase of investigation, the dogs were provided a 3-week wash-out period before the next phase of study began. The first phase of study was with a mixed medium/long-chain triglyceride oil (Form 1); the second phase of study was with a lecithin and sesame oil base (Form 2). The third phase of study was done by providing a chewable form (Form 3). All dogs were fed 100 g of wet dog food immediately after administration of the form being studied at each dosing.

### Hemp Extract Vehicles

Three different forms of the same hemp extract (ElleVet Sciences, Portland, ME) were utilized in the study with Form 1 (Oil A) being a mix of 25% medium-chain triglycerides (Perfect Keto 100% MCT, Austin, TX) and 75% long-chain triglyceride-based organic sesame oil (Jedwards International Inc, Braintree, MA). Each milliliter of the oil contained 28 mg of CBD, 29 mg of CBDA, 1 mg of THC, 0.8 mg THCA, 0.7 mg of cannabigerolic acid (CBGA), and 1.3 mg of cannabichromene (CBC) proven with third-party analysis by a certified ISO/IEC 17025 Laboratory (ProVerde Laboratory, Milford, MA). Form 2 (Oil B) was exactly the same regarding cannabinoid concentration except that 25% of the base oil was from sunflower lecithin (NOW Sunflower Lecithin, Bloomingdale, IL), and the remaining 75% was the same organic sesame oil as Form 1. Form 3 was formulated with the same hemp extract to contain ~5 mg of CBDA and 5 mg of CBD in each soft chew with a similar profile as stated above. For consistency of dose across dogs, each dog was provided two chews of Form 3 every 12 h because more exact dosing in mg/kg was impractical; therefore, the dogs were receiving between 2.0 and 2.3 mg/kg body weight for this particular product. The manufacturing of Form 3 was done using a cold extrusion process after reconstitution of a dough-like consistency using a materials base of peanut butter, glycerin, defatted rice bran, water, molasses dry (cane), glucosamine HCL, dextrose (glucose-dry), sweet potato (powder), hemp oil, brewer's yeast (dehydrated), potato starch, guar gum, rice starch, dehydrated peanut butter, and sorbic acid, in descending order based on weight based on good manufacturing practices.

### Dosing, Blood Draw, and Health Evaluations

The dogs underwent a 24-h pharmacokinetic (PK) assessment with blood draws at 0, 1, 2, 4, 8, 12, and 24 h, with twice-a-day dosing starting the following morning of the 24-h PK experiment with daily dosing (~0.4 mL of oil using a 1 cc syringe or two soft chews) at 7 am and 6 pm for the duration of each phase of the study (2 weeks). For the 24-h PK assessment, dogs had ~4 mL of blood drawn from the jugular or cephalic vein using a 20-gauge needle, which was then placed in a red top coagulation tube and allowed to clot before centrifugation at 3,600 g for 10 min. Serum was collected and immediately frozen at −80°C. At the end of weeks 1 and 2 of twice-daily dosing, dogs were provided their 7 am dose and then had a follow-up blood draw 6 h after dosing to assess mean serum concentration at the halfway point between doses. The dogs also had initial blood draws prior to starting the trial at 6 a.m. on their first day of each arm of the trial, and 6 h after their last 2-week dose to assess serum hepatic enzymes due to prior reports of elevated liver enzymes with daily exposure to hemp-based extracts (1, 2). Dogs were evaluated daily during play time by the staff and were asked to report any somnolence, lethargy, gait abnormalities, ataxia, or behavioral issues.

Serum hepatic biochemical analyses were performed at two time points during the study. A background and a steady state 2-week blood draw were collected to evaluated hepatic function looking at albumin, alanine amino transferase (ALT), alkaline phosphatase (ALP), aspartate aminotransferase (AST), total bilirubin, glucose, and cholesterol (Antech Diagnostics, Irvine, CA).

### Serum Cannabinoid Analysis

Samples were batched within 8 weeks of the end of experimentation on each form and were transported overnight on dry ice to the laboratory for analysis. Cannabinoids analysis was performed by a novel developed method allowing simultaneous measurement of 10 cannabinoids and their metabolites at the Toxicology Research Laboratory, University of Illinois at Chicago. Reference standards for CBD and CBDA were obtained from Restek Corporation (Bellefonte, PT); all other reference and internal standards mentioned below were obtained from Cerilliant Corporation (Round Rock, TX). The concentration of cannabinoids (CBD, CBDA, THC, THCA, CBN, and CBG) and their metabolites (11-OH-THC, 7-OH-CBD, 7-COOH-CBD, and COOH-THC-Glu) in dog serum was determined by high-performance LC-MS/MS (Nexera X2 and LCMS 8050, Shimadzu Corp., Kyoto, Japan).

**Figure 1:**
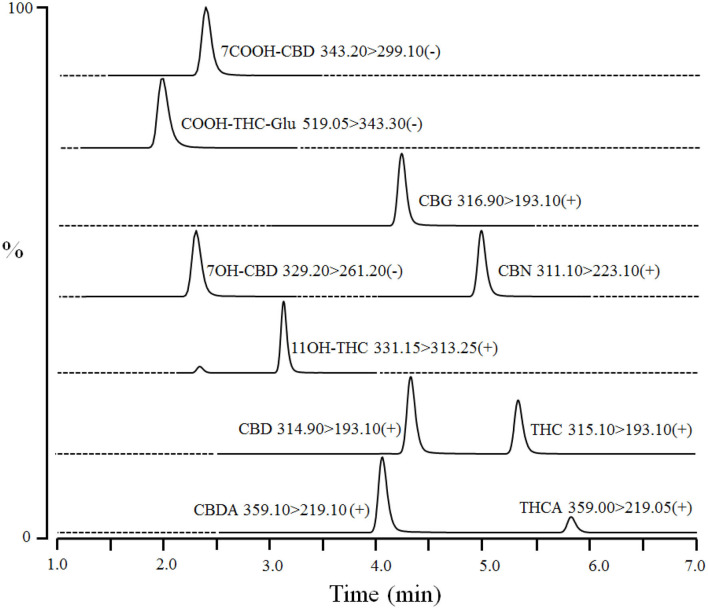
A representative chromatogram of cannabinoids and their metabolites in fortified dog serum at upper limit of quantitation (1,000 ng/mL of each compound). The compound name with corresponding Multiple Reaction Monitoring (MRM) transition and polarity is given in the inset of each chromatogram. Dashed-line represents the diversion of MS detector for a particular MRM transition and time.

Dog serum (40 μL) was mixed with 20 μL of internal standards [100 ng/mL of CBD-d3, THC-d3, 11-OH-THC-d3, and 7-OH-CBD-d5 in water:methanol (50:50)] in a 96-well plate. Proteins were precipitated, and compounds were extracted by adding 80 μL of ice-cold acetonitrile to each sample followed by vortexing (1–2 min) and centrifugation at 4,000 rpm for 10 min at 4°C. The supernatants (100 μL) were mixed with water (100 μL) in another 96-well plate and centrifuged again. The processed samples were injected (10 μL) into Waters Atlantis T3 HPLC column (3 μm 2.1 × 50 mm) coupled to LC-MS/MS. The column was equilibrated with mobile phase A (0.1% formic acid in water) and mobile phase B (acetonitrile) at ratio A: B 50:50 for 0.5 min. The compounds were eluted by a linear gradient from 50% B to 100% B over 6 min, and then held at 100% B for 1 min. Subsequently, the column was re-equilibrated at initial composition for 0.5 min at a flow rate of 0.3 mL/min. An autosampler and column temperature were set at 4 and 30°C, respectively. CBD, CBDA, THC, THCA, CBN, CBG, and 11-OH-THC were detected in electrospray ionization positive mode using transitions *m/z* 314.90 > 193.10, 359.10 > 219.10, 315.00 > 193.10, 359.00 > 219.05, 311.10 >2 23.10, 316.90 > 193.10, and 331.50 > 313.25 at retention times of ~4.3, 4.0, 5.3, 5.8, 5.0, 4.3, and 3.3 min, respectively (Figure 1). In addition, 7-OH-CBD, 7-COOH-CBD, and COOH-THC-Glu were detected in ESI negative mode using multiple reaction monitoring transitions *m/z* 329.20 > 261.20, 343.30 > 299.10, and 519.05 > 343.30 at retention times of ~2.3, 2.2, and 2.0 min, respectively (Figure 1). Interface voltage and temperature were 4 kV and 300°C, respectively. Desolvation line and heat block temperatures were 250 and 400°C, respectively. Nebulizing, heating, and drying gas flow were 2.7, 5, and 5 L/min, respectively.

Concentrations of cannabinoids were calculated by LabSolutions software (Shimadzu Corp., Kyoto, Japan) using a quadratic calibration curve with 1/c^2^ weighing based on relative response (peak area of cannabinoids/peak area of internal standards). The calibration curve range was from 1 to 1,000 ng/mL for CBD, CBDA, THC, THCA, CBN, CBG, and 7-COOH-CBD, and from 2.5 to 1,000 ng/mL for 11-OH-THC, 7-OH-CBD, and COOH-THC-Glu in dog serum.

### Pharmacokinetics and Statistical Analysis

The 24-h pharmacokinetic analysis for each cannabinoid or its metabolite (CBD, CBDA, THC, THCA, CBG, CBN, 11-OH-THC, 7-OH-CBD, 7-COOH-CBD, and COOH-THC-Glu) was performed utilizing a commercial software system that allows for a one compartment model using 5 half-life assumption for mean serum concentration, which is reported (PK solutions 2.0, Summit PK, Montrose, CO). The results generated were time to maximal concentrations (Tmax), maximum serum concentration (Cmax), half-life (T½), area under the curve to the last time point (AUC_0−24_), AUC of the dose extrapolated to infinity (AUC_infin_), mean retention time (MRT), and calculated predicted 5 half-life mean serum concentration (Predict Ave). All of these values and the standard error of the mean are reported with follow-up statistics of each parameter being completed using non-parametric statistics due to the small sample sizes. Friedman's testing with Dunn's *post-hoc* analysis was used to examine differences between the three groups for each pharmacokinetic parameter that was performed. For 7-OH-CBD and 7-COOH-CBD, a Wilcoxon signed rank test was used to assess Form 2 and Form 3 pharmacokinetics; as methodological standards for 7-OH-CBD and 7-COOH-CBD were not available, the time serum from Form 1 was analyzed. To assess the 1- and 2-week serum cannabinoid concentrations post 6 h dosing, a Friedman's test was performed at each week with Dunn's *post-hoc* testing to assess difference between groups at each week, except for 7-OH-CBD and 7-COOH-CBD where differences between Form 2 and Form 3 were assessed using a Wilcoxon signed rank test. A Wilcoxon signed rank test was used to assess the weeks 1 and 2 results for each cannabinoid examined. The hepatic serum chemistry data for all parameters (albumin, AST, ALP, ALT, total bilirubin, glucose, and cholesterol) at weeks 0 and 2 were performed using a Wilcoxon signed rank test. All statistical measures utilized a *p*-value of ≤0.05 to establish statistical significance, except for the weeks 1 and 2 serum concentration data due to multiple comparisons, hence *p* ≤ 0.025 was established after Bonferroni's corrections. All statistical testing was performed with Graphpad Prism 6.0 (Graphpad, LaJolla, CA), and graphs were generated by the same software or Microsoft Excel (Microsoft Inc., Redmond, WA).

## Results

### Twenty-Four Hours Pharmacokinetics

No differences were noted for Tmax, T½, AUC_0−24_, AUC_infin_, MRT or predicted average concentration for all three oral forms of the hemp product when examining CBD pharmacokinetics. The only significant difference in CBD between the three oral delivery forms was with the Form 3 appeared to have a higher Cmax than Form 2, but not Form 1 (*p* = 0.03, see Table 1). CBDA pharmacokinetics showed no differences between any of the forms for Cmax, Tmax, T½, AUC_0−24_, AUC_infin_, MRT or predicted average serum concentration (Table 1). THC concentrations could not be compared over the 24-h time period due to insufficient data for calculation of pharmacokinetics, except for Form 2 which had sufficient data from all dogs to report (Table 1). THCA, however, had ample absorption and serum concentrations over time to assess the differences between all forms showing the only difference in pharmacokinetics was between the Forms 1 and 3 regarding MRT time being increased with the chew (*p* = 0.02; Table 1).

**Table 1:** Mean and SEM (*n* = 6 for all cannabinoids) on serum 24- pharmacokinetics values of CBD, CBDA, THC, THCA, and 7-COOH-CBD.

**CBD (ng/mL)**	**Cmax**	**Tmax**	**T1/2**	**AUC_**0−24**_**	**AUC_**infin**_**	**MRT**	**Predict Ave**
Form 1	145 ± 69	1.5 ± 0.5	4.1 ± 0.7	635 ± 399	656 ± 414	5.2 ± 1.4	53 ± 33
Form 2	124 ± 62	2.0 ± 1.1	4.4 ± 1.4	683 ± 146	707 ± 144	6.5 ± 2.1	63 ± 17
Form 3	226 ± 89^*^	2.5 ± 1.2	3.8 ± 0.3	826 ± 74	845 ± 74	5.3 ± 1.4	70 ± 15
**CBDA (ng/mL)**							
Form 1	383 ± 167	1.0 ± 0.0	4.4 ± 2.7	1,018 ± 308	1,152 ± 451	5.2 ± 3.3	88 ± 41
Form 2	386 ± 213	1.2 + 0.4	4.2 ± 1.0	1,619 ± 898	1,748 ± 855	6.8 ± 2.3	136 ± 66
Form 3	510 ± 350	2.3 + 0.6	2.4 ± 1.1	1,407 ± 585	1,419 ± 591	4.3 ± 1.5	191 ± 158
**THC (ng/mL)**							
Form 1	BQL	BQL	BQL	BQL	BQL	BQL	BQL
Form 2	6 ± 3	1.7 ± 0.5	4.0 ± 3.9	22 ± 9	27 ± 9	6.3 ± 5.7	3.0 ± 0.5
Form 3	BQL	BQL	BQL	BQL	BQL	BQL	BQL
**THCA (ng/mL)**							
Form 1	35 ± 14	1.7 ± 1.2	6.5 ± 5.1	171 ± 57	209 ± 89	9.8 ± 7.6	18 ± 6
Form 2	27 ± 21	2.2 ± 1.0	5.9 ± 2.5	256 ± 114	291 ± 119	8.7 ± 3.7	25 ± 10
Form 3	45 ± 25	3.3 ± 1.0^#^	3.9 ± 0.6	212 ± 69	223 ± 71	6.6 ± 1.7^∧^	25 ± 5
**7-COOH-CBD (ng/mL)**							
Form 1	NA	NA	NA	NA	NA	NA	NA
Form 2	13 ± 2	5.0 ± 0.7	8.4 ± 2.1	159 ± 36	168 ± 41	9.4 ± 1.0	19 ± 4
Form 3	21 ± 2^**^	5.3 ± 0.8	4.8 ± 0.4	188 ± 19	196 ± 22	8.8 ± 0.7	24 ± 2

* *Indicates a statistically different mean Cmax (p < 0.05) than the other groups for CBD*.

** *Indicates a statistically different mean (p < 0.05) than the other group for 7-COOH-CBD*.

∧ *Indicates a significant difference between Form 1 and 3 (p < 0.05)*.

*Cmax, Maximal concentration*.

*Tmax, Time to a maximal concentration*.

*T½, Half-life*.

*AUC_0−24_, Area under the curve 24 h*.

*AUC_infin_, Area under the curve extended to infinity*.

*MRT, Mean retention time*.

*Predict Ave, Predicted 5 half-life average concentration*.

*NA, Not Available*.

*BQL, Below Quantitation Limit*.

**Figure 2:**
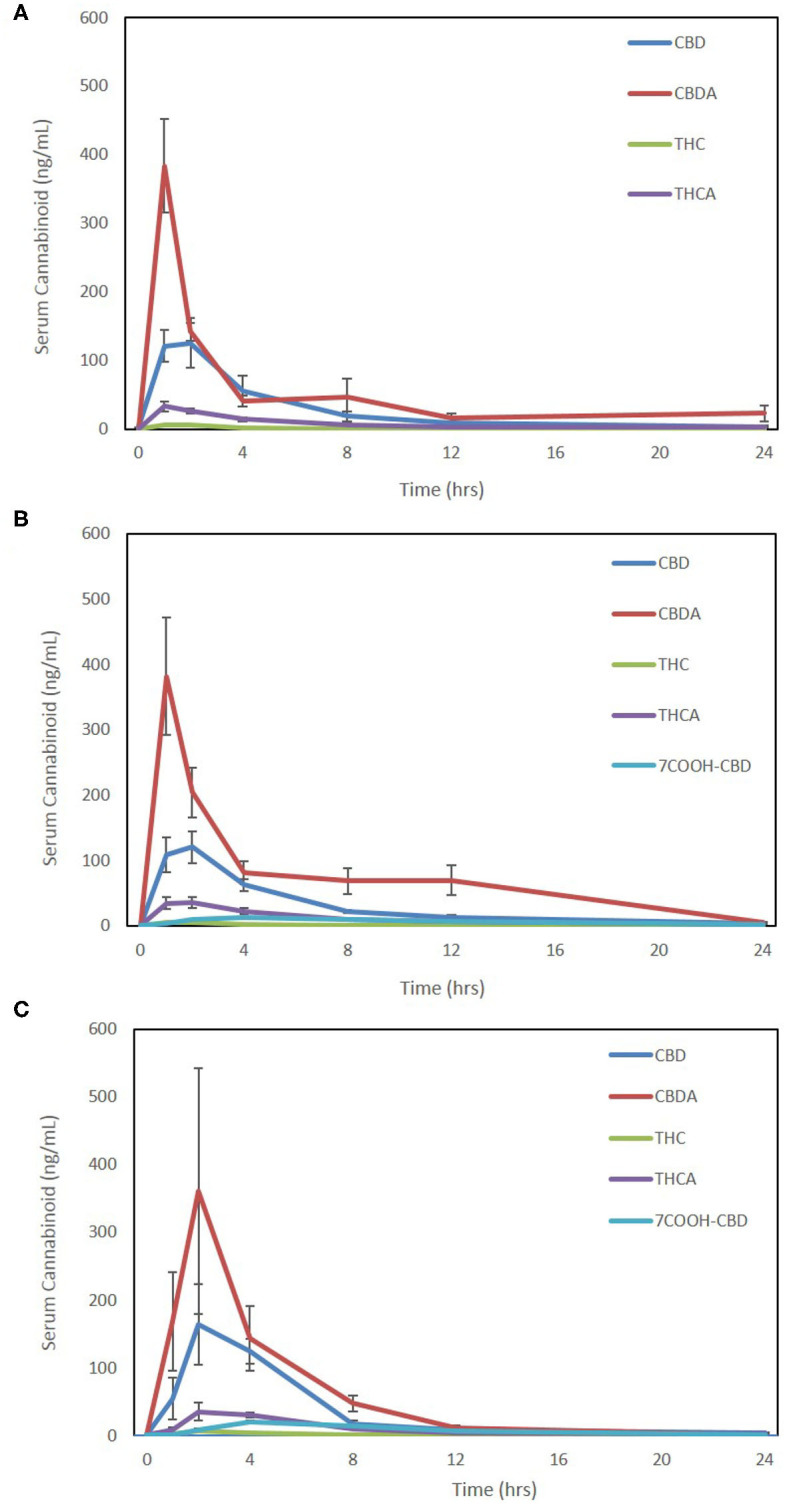
Seven-point 24-h pharmacokinetics of three oral hemp-based forms showing oral absorption kinetics (mean and SEM) of cannabidiol (CBD-blue), cannabidiolic acid (CBDA-red), Δ9-tetrahydrocannabinol (THC-green), tetrahydrocannabinolic acid (THCA-purple), and 7-carboxy- cannabidiol (7-COOH-CBD- light blue for Form 2 and Form 3 only). **(A)** Oral absorption using 25% MCT/75% sesame oil base at 2 mg/kg dosing using a 50 mg/mL hemp cannabinoid preparation (Form A); **(B)** oral absorption using 25% sunflower lecithin/75% sesame oil base at 2 mg/kg dosing using a 50 mg/mL hemp cannabinoid preparation (Form 2); and **(C)** oral absorption using a 10 mg hemp cannabinoid/10 g chew (Form 3) at ~2–2.3 mg/kg body weight.

Due to the absence of the reference standards for 7-OH-CBD and 7-COOH-CBD at the time of analysis of Form 1, it is not possible to compare results for these compounds on this form and the other formulations. The 7-COOH-CBD concentrations could be compared between Form 2 and Form 3, and there were no significant differences in any pharmacokinetic parameters except for Cmax being slightly higher for Form 3 over Form 2 (*p* = 0.02; Table 1). Twenty-four-hour pharmacokinetic curves for CBD, CBDA, THC, THCA, and 7-COOH-CBD are represented in Figures 2A–C.

The metabolites of THC, 11-OH-THC, and COOH-THC-Glu were all below the lower limit of quantitation (1 ng/mL for THC or 2.5 ng/mL for 11-OH-THC and COOH-THC-Glu), except in a few samples where 11-OH-THC was observed at 1 or 2 h (six samples Form 1, four samples Form 2, and two samples Form 3). 7-OH-CBD was undetectable in all samples assessed in Forms 2 and 3. Hence, 24-h pharmacokinetics analysis was not possible for any of these metabolites.

### One- and Two-Week Serum Cannabinoid Concentrations

**Figure 3:**
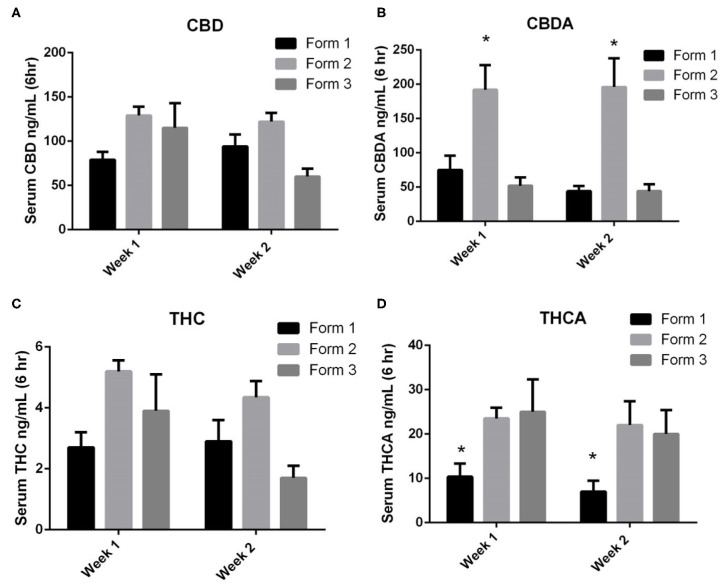
One- and two-week serum cannabinoids concentrations (mean and SEM) 6 h after morning dosing when using Form 1, Form 2, or Form 3 for oral dosing. **(A)** Serum CBD concentrations. No significances noted between formats or times; **(B)** serum CBDA concentrations. *Indicates a significant increase in serum CBDA concentrations for Form 2 as weeks 1 and 2 when compared to Form 1 and Form 3 (*p* < 0.01); **(C)** serum THC concentrations. No significances noted between formats or times; and **(D)** serum THCA concentrations. *Indicates a significant decrease in THCA with Form 1 when compared to Form 2 and Form 3 at both weeks 1 and 2 (*p* < 0.01).

When examining the CBD concentrations in serum at weeks 1 and 2, Form 1 serum concentrations (mean ± SEM) were 79 ± 9 ng/mL and 94 ± 14 ng/mL at weeks 1 and 2. Form 2 serum concentrations (mean ± SEM) were 129 ± 10 ng/mL and 122 ± 10 ng/mL at weeks 1 and 2. Form 3 serum concentrations (mean ± SEM) were 115 ± 28 ng/mL and 60 ± 9 ng/mL at weeks 1 and 2, respectively. There were no statistically significant differences between CBD concentrations between the weeks of exposure or between the different formulations at the two time points (Figure 3A).

CBDA serum concentrations were similar to CBD concentrations at weeks 1 and 2. Form 1 serum concentrations (mean ± SEM) were 75 ± 21 ng/mL and 44 ± 8 ng/ml at weeks 1 and 2. Form 2 serum concentrations (mean ± SEM) were 192 ± 36 ng/mL and 196 ± 42 ng/mL at weeks 1 and 2. Form 3 serum concentrations (mean ± SEM) were 52 ± 12 ng/mL and 44 ± 10 ng/mL at weeks 1 and 2, respectively. Statistically, the serum concentrations of CBDA at both weeks 1 and 2 were significantly higher for Form 2 when compared to Forms 1 and 3 (*p* < 0.01; Figure 3B). When assessing CBD and CBDA across weeks 1 and 2, there were no significant differences in concentrations across any of the formulations, proving relatively similar concentrations over weeks 1 and 2.

THC serum concentrations were far lower than CBD or CBDA concentrations following similar absorption kinetic of CBD. Form 1 serum concentrations (mean ± SEM) were 2.7 ± 0.5 ng/mL and 2.9 ± 0.7 ng/mL at weeks 1 and 2. Form 2 serum concentrations (mean ± SEM) were 5.2 ± 0.4 ng/mL and 4.4 ± 0.5 ng/mL at weeks 1 and 2. Form 3 serum concentrations were 3.9 ± 1.2 ng/mL and 1.7 ± 0.4 ng/mL at weeks 1 and 2, respectively (Figure 3C). No significant differences in concentrations were found between the three different formulations at each time point nor between the weeks 1 and 2 time points.

THCA serum concentrations were higher than THC concentrations and appear to follow kinetics of CBDA absorption and retention. Form 1 serum concentrations (mean ± SEM) were 10.4 ± 1.2 ng/mL and 7.0 ± 1.0 ng/mL at weeks 1 and 2. Form 2 serum concentrations (mean ± SEM) were 23.5 ± 22.0 ng/mL and 25.0 ± 2.2 ng/mL at weeks 1 and 2. Form 3 serum concentrations were 25.0 ± 3.0 ng/mL and 20.0 ± 2.2 ng/mL at weeks 1 and 2, respectively. Both Form 2 and Form 3 had significantly higher THCA concentrations than Form 1 at both weeks 1 and 2 (*p* < 0.01; Figure 3D). There were no significant differences found between weeks 1 and 2 of dosing.

Except for a few samples, COOH-THC-Glu was not detectable in the bloodstream during weeks 1 and 2 testing. 11-OH-THC levels were below the quantitation limit for Form 1, while Form 2 showed two of the six dogs having detectable levels at 2.6 and 6.4 ng/mL at week 1; and three of the six dogs showing detectable concentrations at 4.0, 2.7, and 6.7 ng/mL at week 2. Form 3 showed only one dog at week 2 having an 11-OH-THC concentration higher than the lower limit of quantitation at 2.8 ng/mL.

The CBD metabolites assessed at weeks 1 and 2 across all three oral formulations showed no 7-OH-CBD accumulation in dog serum, with all samples being below the lower limit of quantitation. 7-COOH-CBD concentrations were not determined in Form 1 due to lack of standards at the time of analysis, but 7-COOH-CBD concentrations in Form 2 across the six dogs were 24.5 ± 2.3 ng/mL and 26.1 ± 2.9 ng/mL at weeks 1 and 2, respectively. Serum concentrations of 7-COOH-CBD when using Form 3 were 27.9 ± 4.8 ng/mL at week 1, and 23.7 ± 4.7 ng/mL at week 2. There were no significant differences between weeks 1 and 2 for either dosing form, and no differences in concentrations were observed between the oral forms at each time point.

### Liver Biochemical Analysis and Dog Health

The major enzymes associated with hepatic injury or altered function include ALP, ALT, and AST. We did not observe any significant changes in any of the oral treatments in the serum concentrations of these enzymes prior to beginning of treatment and 2 weeks into twice-daily treatment (Table 2). Serum albumin and cholesterol, which are other parameters of hepatic function, also showed no significant changes due to dosing over the 2-week period (Table 2). Assessment of biliary stasis as measured by total bilirubin shows no significant rises or decreases over the 2-week time period regardless of treatment group (Table 2). Daily assessment of the dogs by the laboratory and animal care personnel showed no abnormalities in behavior or health problems associated with hemp extract administration.

**Table 2:** Mean and standard error of the mean concentrations (*n* = 6) for hepatic biochemistry including ALP, ALT, AST, albumin, total bilirubin, cholesterol, and glucose at 0 and 2 weeks (wk).

**Parameter (Ref. Range)**	**Form 1–0 wk**	**Form 1–2 wk**	**Form 2–0 wk**	**Form 2–2 wk**	**Form 3–0 wk**	**Form 3–2 wk**
ALP (8–114 U/L)	39 ± 3	46 ± 5	37 ± 4	40 ± 4	32 ± 4	35 ± 5
ALT (18–64 U/L)	25 ± 1	23 ± 1	26 ± 2	25 ± 2	26 ± 3	29 ± 4
AST (15–52 U/L)	27 ± 2	24 ± 1	25 ± 2	23 ± 1	24 ± 2	26 ± 2
Albumin (2.9–3.8 g/dL)	3.1 ± 0.5	3.1 ± 0.3	3.1 ± 0.1	3.0 ± 0.1	3.3 ± 0.1	3.4 ± 0.1
Total Bilirubin (0.1–0.4 mg/dL)	0.2 ± 0.1	0.2 ± 0.1	0.2 ± 0.0	0.2 ± 0.0	0.2 ± 0.0	0.2 ± 0.0
Cholesterol (124–334 mg/dL)	210 ± 10	250 ± 29	199 ± 22	223 ± 19	211 ± 20	226 ± 19
Glucose (79–120 mg/dL)	91 ± 4	105 ± 6	100 ± 3	89 ± 6	102 ± 2	91 ± 9

*ALP, alkaline phosphatase; ALT, alanine aminotransferase; AST, aspartate aminotransferase*.

*No significant differences were noted between time points for any treatment group*.

## Discussion

This is the first pharmacokinetic serum assessment of cannabinoids and major metabolites using a whole hemp plant derived extract in dogs, most notably for the major cannabinoid acid forms, CBDA and THCA, which are not routinely assessed. The increase in oral use of whole hemp extract in veterinary and human medicine warrants further investigation into the native acid derivatives of CBD and THC, since existing products that do not undergo heat extraction will contain CBDA and THCA. In much of the prior research on inhaled forms of cannabis, these derivatives were not assessed since the heat-induced decarboxylation of CBDA to CBD and THCA to THC (5, 20). In the human literature, there is very little investigation into CBDA, and it was previously thought that when providing CBDA it either improved the absorption of CBD or rapidly became CBD due to potential for gastric or hepatic conversion (6, 7). Our data shows that the absorption of CBDA does occur in dogs and is absorbed at least twice as well as CBD in 24-h kinetic examination. One limitation of our study design is that we did not do more frequent sampling at 15 and 30 min post-dosing, as it is entirely possible that we missed the Cmax and Tmax for the cannabinioids, particularly since CDBA Cmax is at our first sampling time point. It is unlikely that we missed the Cmax or Tmax for CBD and THC since recent work in the fed canine model shows similar results to ours with Tmax in the 1–2 h range (21); however, the only other pharmacokinetics examining CBDA in humans with more frequent sampling in a fasted model suggesting 0.8–1 h to be the Tmax (22). Therefore, future experiments for CBDA should incorporate these earlier time points in fed or fasted models.

The pharmacokinetics of CBDA over a 2-week period shows nearly equal serum concentrations to CBD, suggesting absorption and retention of oral dosing. More interestingly, is that Form 2, which contains a 25% sunflower lecithin base, showed slightly higher AUC_0−24_ and mean retention time in 24-h pharmacokinetics, which may have translated into the significantly higher weeks 1 and 2 serum concentrations when compared to Form 1 (25% medium chain triglyceride base) or Form 3. The implications of this are unknown from a clinical perspective; however, CBDA may have some unique properties as an anti-inflammatory as well the ability to mitigate nausea through 5HT 1a receptor activation (23–27). CBDA pharma-biological understanding is in its infancy, but it may have equal, if not better absorption when compared to CBD which is worth further investigation.

This investigation is the first of its kind to assess both THC and THCA as minor cannabinoids that are found in most CBD-rich hemp products at lower than 0.3% combined. The quantity of THC and THCA delivered to these dogs was <0.1 mg/kg body weight, which is far lower than prior long-term dosing (25 mg/kg) that resulted in minimal clinical side effects (28). In the prior 52-week study, dogs became habituated to these larger doses and exhibited THC serum concentrations in the tens of ng/mL while our THC concentrations were within 2–5 ng/mL regardless of the form used (28). In addition, no side effects such as ataxia or somnolence was observed in the beagles used in our study at any point during treatment. Interestingly, the THCA concentrations in the dogs were noticeably higher in the 10–25 ng/mL range at weeks 1 and 2 with slightly better retention of THCA in Form 2 and Form 3 treated groups. THCA appearance in the bloodstream at these concentrations may be of therapeutic value since THCA has been suggested to be a non-intoxicating and a neuroprotective cannabinoid, but research into THCA is relatively sparse (15, 29, 30). It must also be recognized that these exploratory investigations into canine serum cannabinoids are not validated procedures (1, 4, 17, 21). Although *R*^2^ values of ≥0.98 were achieved for all cannabinoids when examining goodness of fit calculations, and the accuracy of 75% of calibration standards was within ±15% of the nominal concentrations in our study, there may be higher inconsistencies at data points particularly when many of THC cannabinoids measured were near the lower limit of quantitation (due to very low THC concentrations in the initial formulation products), except for THCA.

Not surprisingly, the 11-OH-THC metabolite, which has been associated with intoxicating effects, showed variable measurable quantities in the 24-h pharmacokinetic profile and could not be universally detected in all dogs at multiple time points, which did not allow for 24-h pharmacokinetic analysis. However, weeks 1 and 2 concentrations were above the lower limit of quantitation (2.5 ng/mL) in a few dogs on Form 2 (*n* = 3) and Form 3 (*n* = 1) with a range of 2.6–6.7 ng/mL. The only other long-term canine assessment of 11-OH-THC suggests that dogs can have concentrations between 6 and 46 ng/mL in their serum and not display outward clinical side effects (26). Humans with 11-OH-THC concentrations in the 2–20 ng/mL concentrations will begin to exhibit cognitive and recall issues when tested without any physiological changes (31, 32). These disparities suggest the possibility of species-dependent sensitivities and metabolism, particularly in light of the evidence that dogs, rats, and humans metabolize CBD and THC differently (33, 34), and due to the insufficient sensitivity of our assay in detecting 11-OH-THC, other methods are necessary to truly evaluate this metabolite (and/or dosing with higher THC levels may be used). The THCA serum concentrations were higher than expected for all forms of treatment when compared to the THC concentrations, yet the mean retention time for THCA was ~70–100% longer than other cannabinoids, which might allow for slightly higher bioaccumulation. Furthermore, we cannot rule out whether THCA is metabolized to THC or eventually to 11-OH-THC, which may be the reasons the occasionally higher 11-OH-THC than THC that was encountered sporadically, though unlikely.

From a metabolic perspective, the metabolism of CBD was also interrogated by assessing the major metabolites identified in humans (7-OH-CBD and 7-COOH-CBD), which are not prevalent in dogs. The levels of 7-OH-CBD could not be measured in our dogs, while 7-COOH-CBD was in the 20–30 ng/mL range as a mean concentration after 1 and 2 weeks of use. These results are dramatically higher than what was recently observed in a study where dogs received 62 mg/kg in a short-term study, suggesting that there may be some adaptation to CBD metabolism or differences in absorption and retention in this study (17). Comparatively, the 7-COOH-CBD is about 1–2% of what has been observed in humans when giving similar doses of CBD (35). This lack of 7-hydroxylation or carboxylation has been observed previously in comparative literature, highlighting that the metabolites are likely to be different and may result in different pharmacological effects across species, which has not been investigated (28). *In-vitro* liver homogenate experiments and metabolite generation suggest hydroxylation and/or glucuronidation at the 4 or 6 position rather than the 7 position (33, 34). If and when standards for such metabolites are developed further, serum analysis may reveal the primary metabolites in dogs, which are currently unknown.

It must also be noted that our experimental design utilized feeding at the time of administration to promote cannabinoid absorption. These are not new findings since older literature suggests that THC uptake is 3- to 4-fold greater in primates when provided with a mixed meal in the form of a cookie (9). More recent data in rats shows that a lipid-based excipient will increase absorption 4-fold in rats (13). Prior work by our group, when using an oil base in fasted dogs at 2 mg/kg body weight as an equal mix of CBD and CBDA, showed a CBD Cmax of ~100 ng/mL, while other groups have published that 10 mg/kg body weight of CBD resulted in a serum Cmax of ~500–600 ng/mL (1, 21). More recent canine data regarding provision of 2 mg/kg as an equal mix of CBD and CBDA in a soft chew showed serum Cmax of ~300 ng/mL of CBD, further suggesting promotion of absorption with a mixed-meal soft chew (4). The use of the soft chew matrix may have produced the higher maximal concentrations observed; however, these dogs were fasted before administration of the soft chew with no follow-up feeding, which may have led to the quickened half-life observed (4). The current study utilized oils (Forms 1 and 2) or soft chews (Form 3) with a small mixed meal (110 g of canned wet dog food), which showed similar Cmax, Tmax, T½ life, and AUC for each form of treatment used; with trends toward Form 3 showings slightly better absorption of CBD in the pharmacokinetic models based on increased Cmax, while Form 2 has the highest weeks 1 and 2 mean CBD and CBDA concentrations.

The use of cannabinoid-rich hemp-based oils for health and disease is in its infancy in human and veterinary clinical medicine. CBD use is proving efficacious in seizure disorders, multiple-sclerosis-based spasm and pain, oncologic side effects of chemotherapy, with a more recent indication in anxiety and schizophrenic disorders using single or twice-a-day dosing between 5 and 20 mg/kg per day in humans (15, 36–38). In veterinary medicine, three clinical studies have suggested potential use of CBD-rich hemp oils in seizure and osteoarthritis disorders in canines (1–3). The only side effects noted in these studies were rises in the alkaline phosphatase enzyme presumably due to hepatic cytochrome p450 enzyme upregulation. These hepatic effects are observed rather universally at doses of 10 mg/kg or higher in dogs (19). The use of 2 mg/kg of a CBD/CBDA-rich cannabinoid oil did show some modest rises in ALP in a geriatric population (1). However, the current study, and a prior study in healthy young beagles, have not shown any rises in any enzymes outside of normal reference ranges associated with the hepatobiliary system, signifying that the CBD-rich hemp product used in this study at this concentration is likely to be safe (4), particularly in light of the recent long-term GW pharmaceutical study in dogs using 10–100 mg/kg of CBD daily for 39 weeks proving safe with a no observed adverse effects limits being set at 100 mg/kg in that toxicity trial (39).

Overall, this study is the first to show that CBDA and THCA are readily absorbed and retained in dogs with some differences observed in CBDA absorption and/or retention depending on the medium used to deliver the oral treatment. The finding of mid dosing concentrations of 75 ng/mL of CBD and CBDA or greater suggests potential for therapeutic use when delivered at 1 mg/kg body weight for each of these cannabinoids with food. A more interesting finding is the retention of THCA in the serum of between 10 and 25 ng/mL. The exact functions of CBDA and THCA physiologically suggest similar therapeutic benefits to CBD that may have the potential to work synergistically with CBD. These synergistic properties known as the “entourage effect” are currently thought to be the primary reason that lower CBD whole hemp extract dosing can be therapeutic when compared to purified CBD (40, 41). Although the hemp extract product used in this study appears to be generally safe, the results of this study cannot be translated to other products due to differences in variable absorption dependent on carrier oils, and cannabinoid and terpene profiles. This fact makes recommendations of CBD-rich hemp products globally tenuous, and veterinarians should become versed on products available before using them clinically.

## Data Availability Statement

The raw data supporting the conclusions of this article will be made available by the authors, without undue reservation.

## Ethics Statement

The animal study was reviewed and approved by University of Florida Institutional Animal Care and Use Committee.

## Author Contributions

JW, WS, and SC were responsible for conceptualization and study design. All authors were involved in acquisition and analysis of portions of the data, involved in manuscript preparation/revisions, and approved this manuscript before submission. The manuscript was drafted by JW.

## Conflict of Interest

JW is currently a paid consultant for Ellevet Sciences, and SC is currently an employee of Ellevet Sciences. The funding for this study was provided to JW at the University of Florida by Ellevet Sciences. The funder was involved in the study design under the guidance of author JW. The funder was not involved in the collection, analysis, interpretation of data, the writing of this article, or the decision to submit it for publication. The remaining authors declare that the research was conducted in the absence of any commercial or financial relationships that could be construed as a potential conflict of interest.

## Abbreviations

CBD: Cannabidiol
CBDA: Cannabidiolic Acid
7-OH-CBD: 7-Hydroxycannabidiol
7-COOH-CBD: 7-Nor-7-carboxycannabidiol
CBG: Cannabigerol
CBN: Cannabinol
THC: (-)-Δ^9^-Tetrahydrocannabinol
THCA: Δ^9^-Tetrahydrocannabinolic Acid
11-OH-THC: (±)-11-Hydroxy-Δ^9^-THC
COOH-THC-Glu: (+)-11-nor-9-Carboxy-Δ^9^-THC glucuronide.
